# Detection and visualization of complex structural variants from long reads

**DOI:** 10.1186/s12859-018-2539-x

**Published:** 2018-12-21

**Authors:** Zachary Stephens, Chen Wang, Ravishankar K. Iyer, Jean-Pierre Kocher

**Affiliations:** 1 Coordinated Science Lab, University of Illinois at Urbana-Champaign, Urbana, IL USA; 2 Mayo Clinic, Rochester, MN USA

**Keywords:** Structural variation, Complex rearrangement, Long read

## Abstract

**Background:**

With applications in cancer, drug metabolism, and disease etiology, understanding structural variation in the human genome is critical in advancing the thrusts of individualized medicine. However, structural variants (SVs) remain challenging to detect with high sensitivity using short read sequencing technologies. This problem is exacerbated when considering complex SVs comprised of multiple overlapping or nested rearrangements. Longer reads, such as those from Pacific Biosciences platforms, often span multiple breakpoints of such events, and thus provide a way to unravel small-scale complexities in SVs with higher confidence.

**Results:**

We present CORGi (COmplex Rearrangement detection with Graph-search), a method for the detection and visualization of complex local genomic rearrangements. This method leverages the ability of long reads to span multiple breakpoints to untangle SVs that appear very complicated with respect to a reference genome. We validated our approach against both simulated long reads, and real data from two long read sequencing technologies. We demonstrate the ability of our method to identify breakpoints inserted in synthetic data with high accuracy, and the ability to detect and plot SVs from NA12878 germline, achieving 88.4*%* concordance between the two sets of sequence data. The patterns of complexity we find in many NA12878 SVs match known mechanisms associated with DNA replication and structural variant formation, and highlight the ability of our method to automatically label complex SVs with an intuitive combination of adjacent or overlapping reference transformations.

**Conclusions:**

CORGi is a method for interrogating genomic regions suspected to contain local rearrangements using long reads. Using pairwise alignments and graph search CORGi produces labels and visualizations for local SVs of arbitrary complexity.

## Introduction

The detection and annotation of structural variants (SVs), including large insertions, deletions, or inversions of genomic sequence, is central to the study of human genetics as these events are frequently found to be associated with natural variation, disease phenotypes, and drug metabolism [1–3]. SVs remain challenging to detect with high sensitivity in part due to the limited ability of short-read sequencing data to span large events or to identify breakpoint coordinates with high confidence. In addition, SV breakpoint positions are often non-uniquely defined, due to surrounding repetitive sequence elements. These problems are exacerbated for *complex* structural variants, which we define as SVs with clusters of multiple breakpoints representing insertions, deletions and inversions which may be nested or overlapping. These complex rearrangements can arise from serial replication slippage, fork stalling and template switching, and other mechanisms [4, 5]. Though these types of SVs are rarer and more difficult to detect than single nucleotide variants or simple deletions/duplications, their potential role in underlying disease in somatic and germline genomes has garnered significant interest [6–8]. Motivated by this, we seek to detect and visualize fine-grain patterns of complex SVs that may be misidentified or missed entirely by existing methods [9, 10].

Long-read sequencing platforms, such as those by Pacific Biosciences (abbreviated as PacBio) or Oxford Nanopore, offer greater statistical power for detecting SVs with high confidence. Longer reads have higher “mappability,” that is, they can be uniquely aligned to a greater proportion of repetitive regions of the human reference genome, where SVs are disproportionately found [11, 12]. In addition, longer reads are more likely to span multiple breakpoints of a complex event, providing stronger evidence for rearrangements that involve several regions. In recent work, Huddleston et al. [13] showcased the advantage of longer reads by identifying SVs in a haploid human genome, the majority of which were reported to have been missed by the analysis of the 1000 Genomes Project using short-read data [13]. Similarly, Sedlazeck et al. [14] demonstrated the ability of long reads to detect complex SVs from both simulated long reads, and PacBio data from a breast cancer cell line (where they identified inverted duplications and inversions with flanking deletions).

As long-read sequencing technologies continue to become more affordable and efficient it will become crucial to have robust tools that leverage their enhanced ability to unravel complex SVs. To this end, we have developed CORGi (COmplex Rearrangement detection with Graph-search), a tool to detect and plot local genomic rearrangements of arbitrary complexity from long read sequencing data. CORGi can articulate many fine-grain patterns that are frequently found to flank larger rearrangements, providing breakpoint coordinates with base-pair resolution when possible. CORGi works by exhaustively realigning reads suspected to contain evidence for a SV (i.e. *soft-clipped* sequence, or indels in CIGAR strings) against the surrounding reference region, and constructing a directed graph representing a collection of possible rearrangements that are supported by the reads. The highest-scoring structure is found via graph search, and from this graph CORGi automatically produces an event label and output report describing the observed SV pattern.

CORGi takes a read alignment as input, where complex SVs present as groups of breakpoints across multiple loci, or as a cluster of breakpoints in close proximity. In this work, we restrict our attention to breakpoints clustered around a single locus (referred to as local rearrangements).

To validate CORGi we created synthetic datasets and inserted a range of simple and complex SVs to assess breakpoint detection performance. In addition, we apply CORGi to PacBio long reads from NA12878 germline and perform concordance analysis against Illumina TruSeq Synthetic Long Reads (formerly known as “Moleculo,” and will be referred to as such for the remainder of this manuscript) from the same sample. We show that CORGi has high sensitivity for simple and flanked SVs in synthetic data, and performs well on complex local events within a certain size and read error rate. Specifically, we find that for SVs with many breakpoints CORGi’s default parameters are best suited for reads with error rates ≤5*%*. Our results on real data from NA12878 highlight the ability of CORGi to identify SVs with ambiguous breakpoints as well as SVs with flanking insertions, deletions, or other structural complexities.

## Methods

**Fig. 1:**
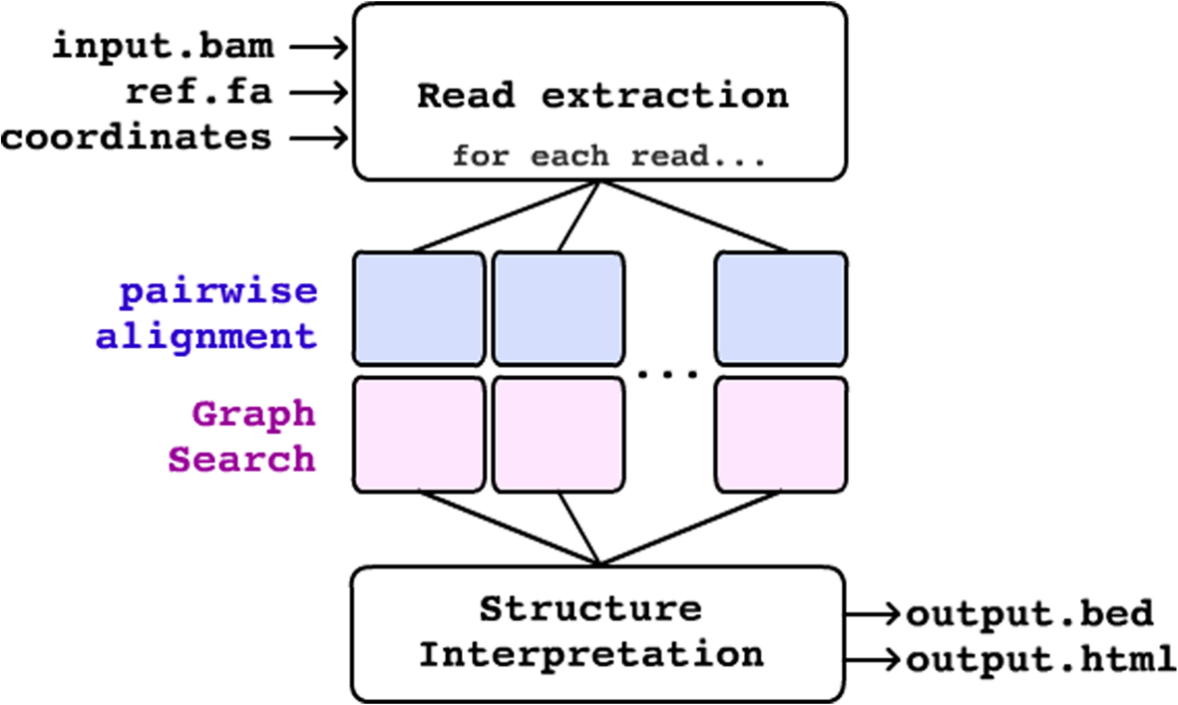
Overview of CORGi

CORGi takes as input a long-read alignment in SAM/BAM format [15], as well as the genomic coordinates suspected to contain SV breakpoints. If sufficient evidence for a SV is found then its coordinates are output in BED format (Fig. 1). In addition, a HTML report containing interactive plots is produced for each high-scoring rearrangement.

### Pairwise alignment

**Fig. 2:**
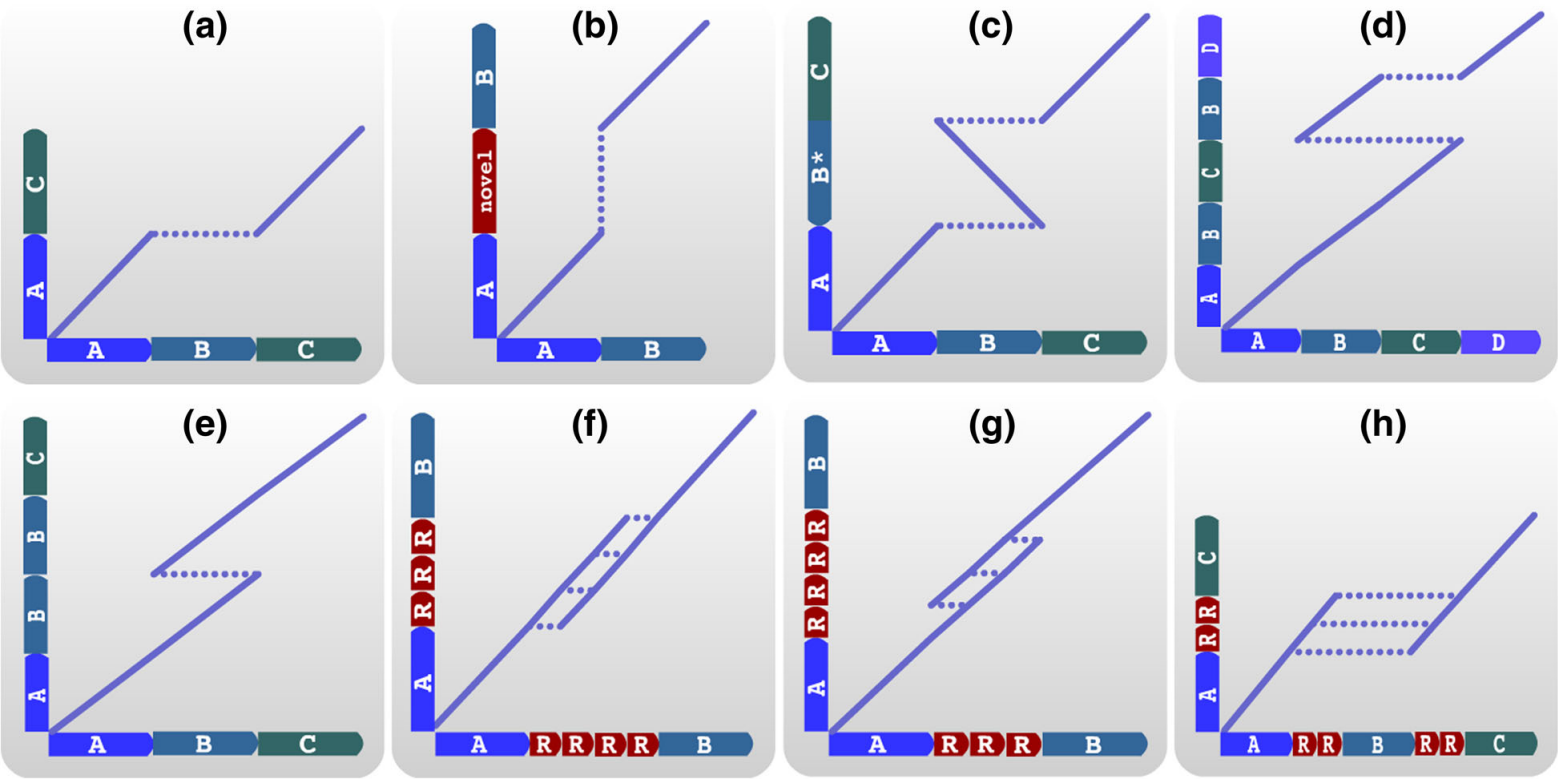
Pairwise alignments of sequences containing structural variation (vertical axis) against a reference sequence (horizontal axis). Diagonal lines indicate alignment matches; horizontal gaps correspond to deletions with respect to the reference; and vertical gaps correspond to insertions with respect to the reference. **a** Simple deletion, **b** simple insertion, **c** simple inversion, **d** dispersed duplication, **e** tandem duplication, **f** loss of repetitive sequence, **g** gain of repetitive sequence, **h** deletion with breakpoints in repetitive sequence. Note that in the last three examples there are multiple pairs of breakpoint coordinates that would result in the same observed rearrangement

CORGi begins by extracting reads that overlap the specified coordinates and contain either soft-clipped sequence, or a large insertion or deletion in its CIGAR string. Each read is then exhaustively aligned against the local reference region using BLASTN [16], producing a collection of pairwise alignment matches. Our method builds on BLAST output by aggregating alignment matches that are supported by contiguity in the long read data, thus inferring how the reference regions may have been rearranged in the presence of an SV (similar in premise to [17]). Each basic SV type is characterized by a specific pattern in the pairwise alignment (Fig. 2), and complex events can be described by a chain of these simple patterns.

### Graph search

**Fig. 3:**
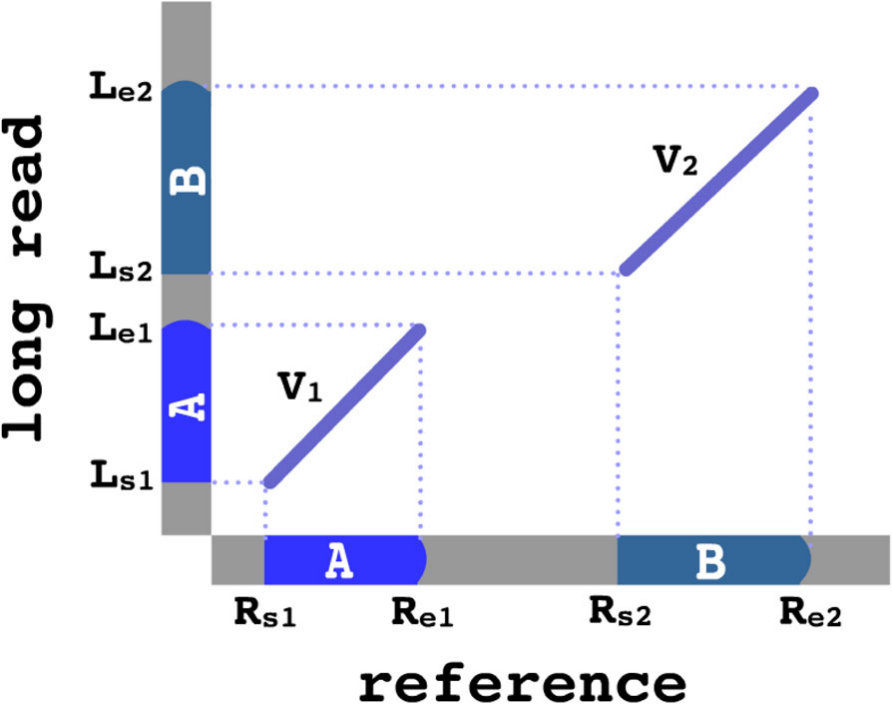
Example vertices *V*_1_, *V*_2_ and their associated read and reference coordinates. In this case *l*(*V*_2_)=*L*_*e*2_−*L*_*s*2_ and *d*(*V*_1_,*V*_2_)=*L*_*s*2_−*L*_*e*1_, thus *E*_12_=*L*_*e*2_+*L*_*e*1_. For simplicity *V*_1_ and *V*_2_ are shown to represent forward alignment matches, but reverse-complement matches (e.g. inversions) are handled similarly

Next, we construct a graph, *G*=(*V,E*), from the pairwise alignments where each vertex *V*_*i*_ corresponds to an alignment match (i.e. the diagonal lines as shown in Fig. 2). The edges *E*_*ij*_ between each pair of vertices represent the hypothesis that the two reference regions associated with *V*_*i*_ and *V*_*j*_ are in fact contiguous in the sample’s genome (and thus in the long reads). The edges are weighted using a scoring function: *E*_*ij*_=*l*(*V*_*j*_)−*d*(*V*_*i*_,*V*_*j*_), where *l*(*V*_*j*_) denotes the number of read coordinates spanned by *V*_*j*_ but not by *V*_*i*_, and *d*(*V*_*i*_,*V*_*j*_) denotes the distance, in coordinates along the read, between *V*_*i*_ and *V*_*j*_. An example is shown in Fig. 3. This scoring function was chosen to reward connections between alignment matches that span a significant amount of sequence content in the long read (rewarding connections in proportion to how much of the observed DNA sequence they cover), while penalizing gaps that are not well supported by contiguity in the reads. After constructing this graph, CORGi derives SV calls from *G*^∗^, the highest scoring subgraph of *G*, which is found by solving: $G^{\ast } = \arg \underset {G}{\max } \underset {i,j \in G}{\sum } E_{ij}$.

Finding *G*^∗^ is the most computationally intensive step, so we apply several heuristics to keep *G* sparsely connected, thus improving computational performance. These optimizations are needed because exhaustively aligning long reads to repetitive reference regions often yields a large number of spurious matches that should be pruned. Specifically, edges *E*_*ij*_ are only connected if *V*_*i*_ and *V*_*j*_ match a sensible rearrangement pattern: For a given pair of vertices, *V*_1_ and *V*_2_, with reference coordinates (*R*_*s*1_,*R*_*e*1_), (*R*_*s*2_,*R*_*e*2_) and long read coordinates (*L*_*s*1_,*L*_*e*1_), (*L*_*s*2_,*L*_*e*2_) (as shown in Fig. 3), a connection will only be formed if one of the following conditions are met:

- *Case 1: Perfect junction:* *L*_*e*1_=*L*_*s*2_
- *Case 2: Novel insertion:* *L*_*s*2_−*L*_*e*1_≤*C*_*N*_, where *C*_*N*_ is a constant parameter specifying the maximum permitted size of a novel insertion (default: 500 bp).
- *Case 3: Ambiguous deletion* (e.g. *copy loss*): *L*_*s*2_<*L*_*e*1_, *R*_*s*2_>*R*_*e*1_ and *L*_*e*2_−*L*_*e*1_≥*C*_*A*_, where *C*_*A*_ is a constant parameter specifying the minimum span of read coordinates that must be uniquely covered by *V*_2_ (default: 1 kb).
- *Case 4: Ambiguous insertion* (e.g. *copy gain*): *L*_*s*2_<*L*_*e*1_, *R*_*s*2_<*R*_*e*1_ and *L*_*e*2_−*L*_*e*1_≥*C*_*A*_, where *C*_*A*_ is the same parameter from case 3.

**Fig. 4:**
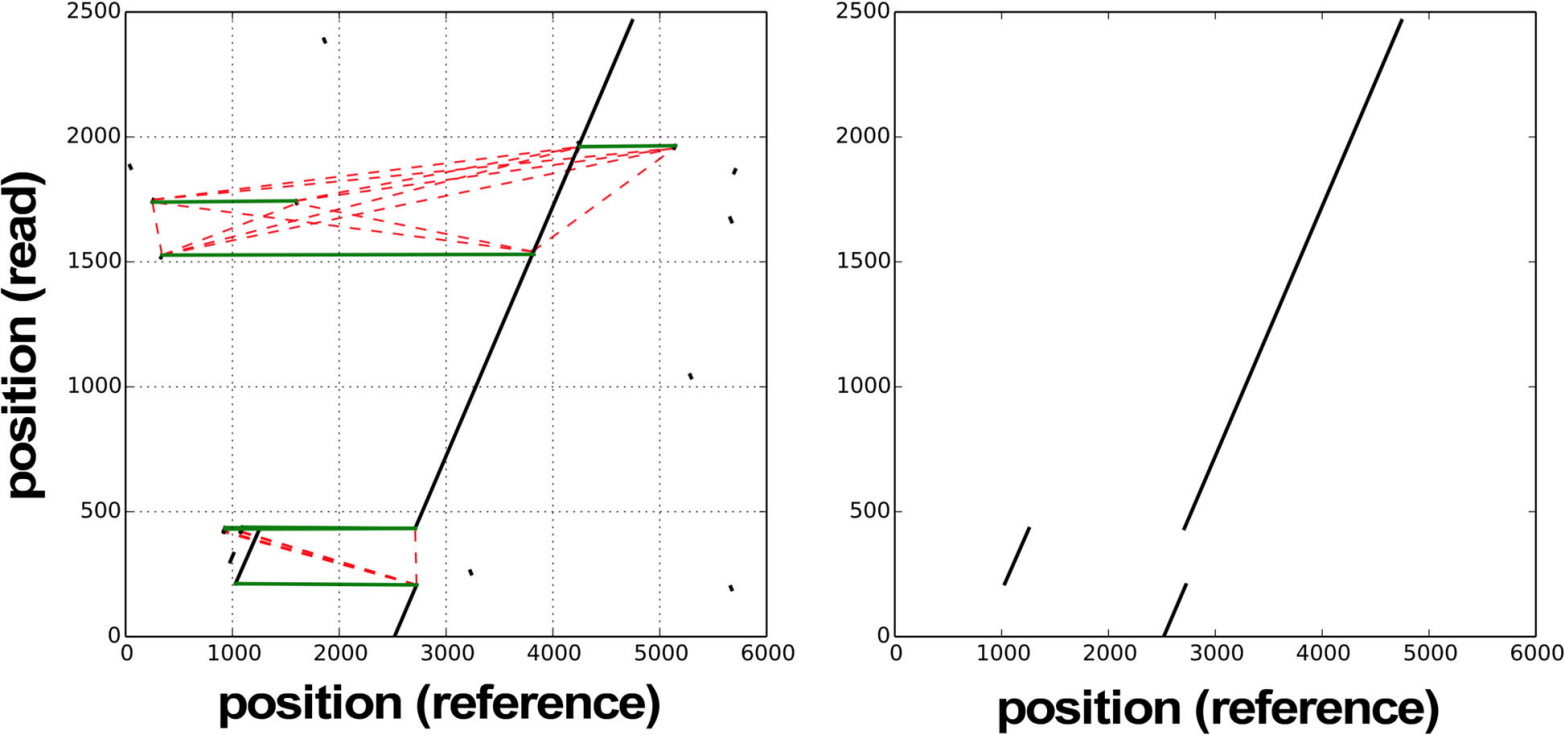
**(Left:)** An example of a graph *G* derived from a pairwise alignment. Perfect junctions are shown as green connections between alignment matches, novel insertion connections are shown as red dashed lines. **(Right:)** The highest scoring subgraph *G*^∗^ that contains the pattern of a dispersed duplication

By imposing these constraints we guarantee that *G* is a directed acyclic graph, as its vertices are only connected such that they make forward progress along the read (Fig 4). Because of this, *G*^∗^ can be found efficiently via dynamic programming. The default values for parameters *C*_*N*_ and *C*_*A*_ were chosen based on preliminary analysis of complex SVs in NA12878. Specifically, *C*_*N*_=500 was chosen to be a reasonable maximum length for inserted sequence flanking a breakpoint. Similarly, *C*_*A*_=1000 was chosen as the maximum allowable size of local homology to report as flanking a breakpoint.

### Structure interpretation

*G*^∗^ is found for each extracted read, and the resultant graphs are aggregated and sorted by the number of reads that support each rearrangement structure. Structures that are not supported by a user-specified minimum number of reads are discarded. The well-supported graphs are then used to enumerate and label the reference regions that participate in the rearrangement. Each region is labeled with a letter, as shown in Fig. 2. As an example, if the SV involved three reference regions **ABC**, a simple deletion of the middle region **B** would yield **AC**, a simple duplication would yield **ABBC**, and so on.

**Fig. 5:**
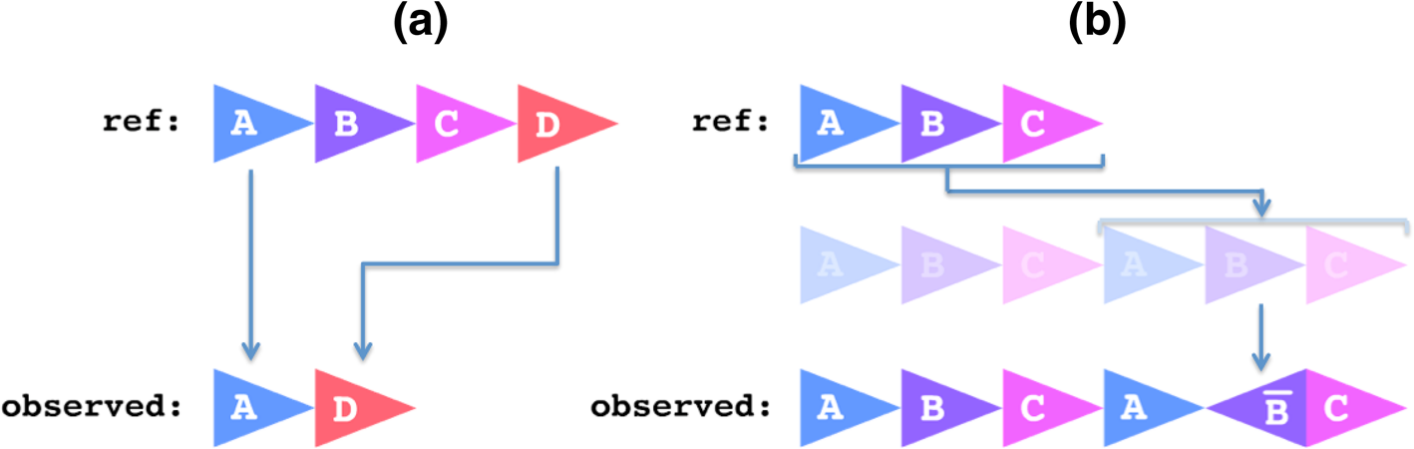
Example rearrangement descriptions. **a** A block deletion of two regions **BC**. **b** A duplication of **ABC** with nested inversion $\overline {\mathbf {\texttt {{B}}}}$

From this string representation, the rearrangement is classified by a labeling algorithm that generates a description of the event based on the minimum number of reference alterations required to match the observed sequence. For this, we use a breadth-first search, successively applying all possible deletions, duplications, and inversions to the reference until the observed pattern is reproduced. Tracing back the path of alterations yields the final output label describing the event. This process is comparable to computing the edit distance between two strings while also allowing contiguous blocks of length ≥2 to be inserted or deleted. For example, **ABCD** →**AD** would be considered a single deletion of **BC**, and **ABC** →**ABCA**$\overline {\mathbf {\texttt {{B}}}}$**C** would be considered two operations: A duplication of **ABC** followed by a nested inversion **B** →$\overline {\mathbf {\texttt {{B}}}}$ (Fig. 5). The search space increases exponentially with the number of string operations, thus labeling very complex rearrangements can be computationally prohibitive. To address this, we add a heuristic to the breadth-first search such that duplications and deletions are only attempted if it brings the total occurrences of the regions involved closer to the final goal. Additionally, the reference string and observed rearrangement string are compressed before labeling if they share any substrings, reducing the search space further.

After a label is produced, SV coordinates are output and each well-supported rearrangement is compiled into an HTML report. Interactive plots are generated using the Bokeh plotting library [18], allowing users to zoom into areas of interest or mouse-over regions for more information on their coordinates and sequence content.

## Results and discussion

To verify that CORGi is capable of correctly deducing SV structures, we created synthetic long read data using the NEAT read simulator [19]. Following this analysis, we apply CORGi to a subset of sites in NA12878 germline using PacBio and Moleculo long reads.

### Simulated data

To create synthetic SVs we injected a range of SV types and SV sizes into a subset of human chr1 and generated long reads using the NEAT read simulator. The reads were then aligned with BWA-MEM [20] to chr1, and the resultant BAM was used as input to CORGi. In our simulation we used an average coverage of 20x, a fixed read length of 5000. We swept over multiple values of read error rates, from 1 to 10*%* (using a sequencing error model derived from PacBio reads, which predominantly injects indel base-calling errors). The specific sites for inserting SV breakpoints were chosen such that they included both repetitive and non-repetitive regions, measured using linguistic sequence complexity. The low complexity sites were chosen to reflect the observation that SVs tend to be found disproportionately in such regions.

In total, we generated 5720 synthetic datasets across 13 event types (Table 1): Using 11 event sizes, 4 read error rates, and 10 positions along chr1 (13 x 11 x 4 x 10 = 5720). For 1139 (19.9*%*) of these events, CORGi did not report any rearrangement that passed default quality thresholds. Nearly every case that failed to produce an SV call was simulated with 10*%* sequencing errors, suggesting that the default parameter values chosen for CORGi’s filtering and read alignment steps are ill-suited for reads with such high error rates. The remaining 4581 (80.1*%*) datasets that yielded output were assessed for accuracy by comparing the structure of the SV called by CORGi with what was injected into the reads. Of these, 4105 (89.6*%*) had all of their breakpoints correctly reported to within ≤10 bp of their inserted position, with performance ranging from virtually 100*%* accuracy for simple events at low error rates, down to ∼20*%* for complex nested events at high error rates.

**Table 1:** Simulated SV types

Simple events	Flanked events	Nested events
Deletion (DEL)	DEL + insertion	TDUP with nested DEL
Tandem duplication (TDUP)	TDUP + insertion	TDUP with nested TDUP
Dispersed duplication (DDUP)	DDUP + insertion	TDUP with nested INV
Inverted duplication (IDUP)	IDUP + insertion	
Dispersed inverted duplication (DIDUP)	DIDUP + insertion	

**Fig. 6:**
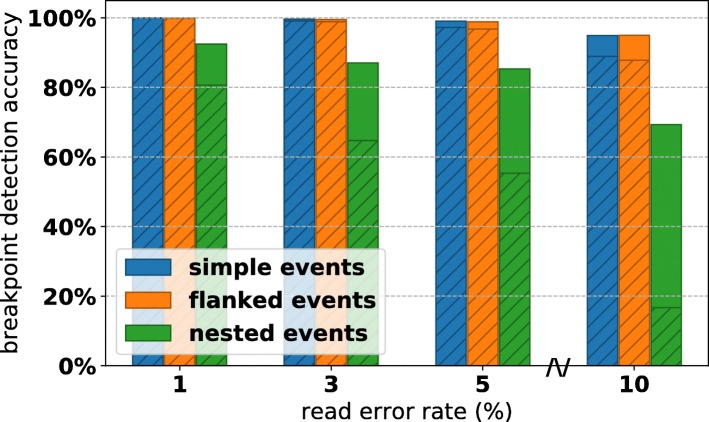
Breakpoint detection accuracy for all simulated data, stratified by read error rate. The height of the bars corresponds to the proportion of individual breakpoints that were found by CORGi within ≤10 bp, the shaded regions correspond to the proportion of simulated SVs that had all of their breakpoints correctly reported

**Fig. 7:**
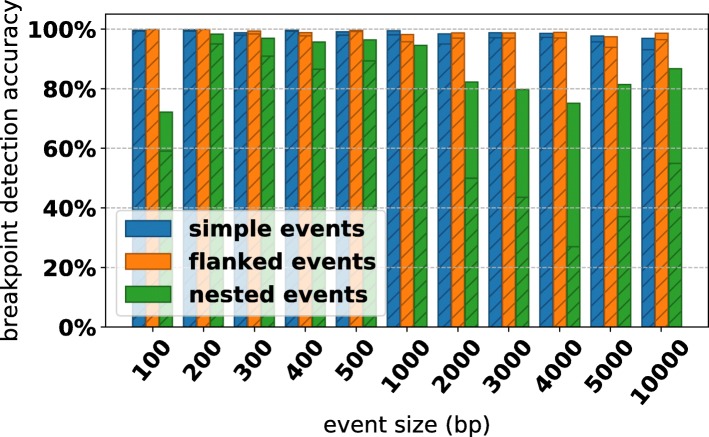
Breakpoint detection accuracy for all simulated data, stratified by the size of the rearrangement. The height of the bars corresponds to the proportion of individual breakpoints that were found by CORGi within ≤10 bp, the shaded regions correspond to the proportion of simulated SVs that had all of their breakpoints correctly reported

Detection performance was stratified by event type, event size, and simulated read error rate (Figs. 6 and 7). We observe that for error rates of 1−5*%*, we achieve >99*%* accuracy for simple SVs and SVs flanked with small novel insertions, falling to ∼90*%* accuracy at error rate 10*%*. Complex events proved more difficult, with about 80*%* of nested events having all of their breakpoints recovered at low error rates, and as low as 20*%* accuracy at high error rates. However, if we consider the proportion of total breakpoints in complex events, we see that CORGi is correctly identifying a majority of the positions where a rearrangement is occurring, even for complex events at 10*%* error. This suggests that in many such cases some component of the structure is correctly identified, but the entire nested event is not reported in full detail.

In Fig. 7, we see that the detection of simple and flanked events appears to be unaffected by the size of the duplicated or deleted regions. Interestingly, the detection accuracy for nested events peaks around 200-500 and decreases as the events get larger. We suspect that the dip in detection accuracy around SV size 4000-5000 bp may be related to the read length chosen for simulation, and in ongoing work we are investigating this by generating synthetic data with a distribution of different read lengths. Additionally, CORGi’s detection accuracy for very small nested events (involving duplications ≤100 bp) is surprisingly low, suggesting that default parameters, such as the minimum number of read positions that need to be anchored in each region involved in the rearrangement, are too large to detect such events with high sensitivity. It may be possible to address this by rerunning the analysis of a site with more lenient settings if CORGi fails to detect an event the first time around. However, this approach is expected to have diminishing returns as decreasing the filtering parameters is likely to increase the number of false positive breakpoint calls.

### Real data: NA12878 Germline

Next, we considered sites in NA12878 suspected to contain SVs based on the presence of soft-clipping or indels (≥5bp) in CIGAR strings. For this analysis we used error-corrected PacBio reads from the Genome in a Bottle Consortium (ftp://ftp-trace.ncbi.nih.gov/giab/ftp/data/NA12878/NA12878_PacBio_MtSinai/) [21], as well as Moleculo long reads (ftp://ftp.1000genomes.ebi.ac.uk/vol1/ftp/phase3/integrated_sv_map/supporting/NA12878/moleculo) provided as a BAM file from the 1000 Genomes Project [22]. Both sets of reads were aligned to human reference hs37d5 using BWA-MEM. After extracting reads with soft-clipped regions or large indels we identify reference coordinates where a boundary between matching sequence and clipped/inserted/deleted sequence is supported by at least five reads.

CORGi was run on all such sites in NA12878, of which 1894 were found to have strong support for a local rearrangement in either the PacBio or Moleculo alignment. In 1519 of these sites (80.2%), CORGi reported the exact event (identical breakpoint locations and identical rearrangement structure) in both the PacBio and Moleculo data. At 155 other sites (8.2%) the reported SVs were found to be approximately equivalent (identical structure but breakpoint positions that differ by at most 10 bp). At the remaining 220 sites (11.6%) the results did not match, either due to insufficient evidence in one of the alignments, or reported structures that did not match. Further inspection of these 220 regions revealed that the cause of mismatch was often due to either lack of coverage in one of the alignments, or lack of consensus on breakpoint positions due to inopportunely placed sequencing errors (the rates of which were observed to be about ∼1*%* in Moleculo, mostly substitutions, and ∼2*%* in PacBio, mostly insertions). By tuning the parameters of our algorithm for each site (e.g. loosening or increasing filter requirements, such as the minimum number of reads needed to support each breakpoint, or the minimum number of coordinates that are required to overlap each side of a breakpoint) we were often able to “recover” missed SV calls, but such cases were not included in our results.

1389 of the 1894 regions were found to intersect with high-confidence SV calls reported by LUMPY [23]. The LUMPY calls were derived from short-read data, and were found by the authors to be supported by both PacBio and Moleculo alignments. These short-read SV calls provide us with a collection to regions to interrogate with CORGi, potentially identifying complex SVs that were previously labeled as simple events. We observe higher concordance between CORGi’s PacBio and Moleculo results in these regions, with 1301 out of 1389 (93.7%) exactly or approximately matching. This suggests that the LUMPY calls predominantly reside in genomic regions less susceptible to issues of coverage and mappability.

**Fig. 8:**
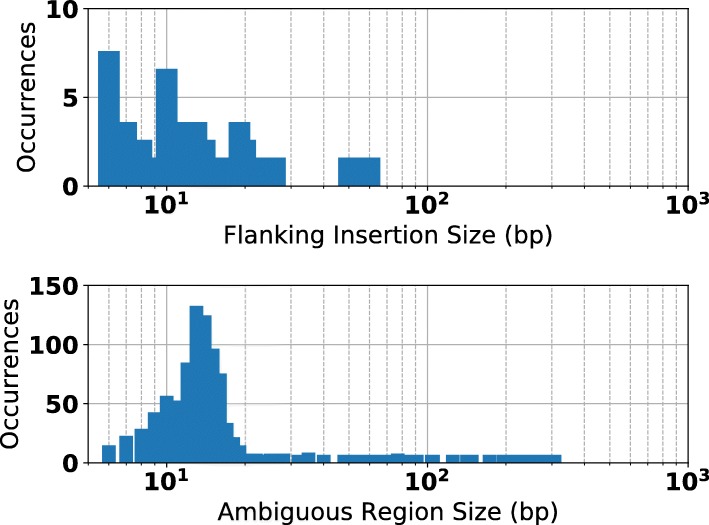
**(Top):** Flanking insertion sizes observed to accompany deletions. **(Bottom):** Ambiguous region sizes observed to surround breakpoints of deletions that could not be pinned to precise genomic coordinates

**Fig. 9:**
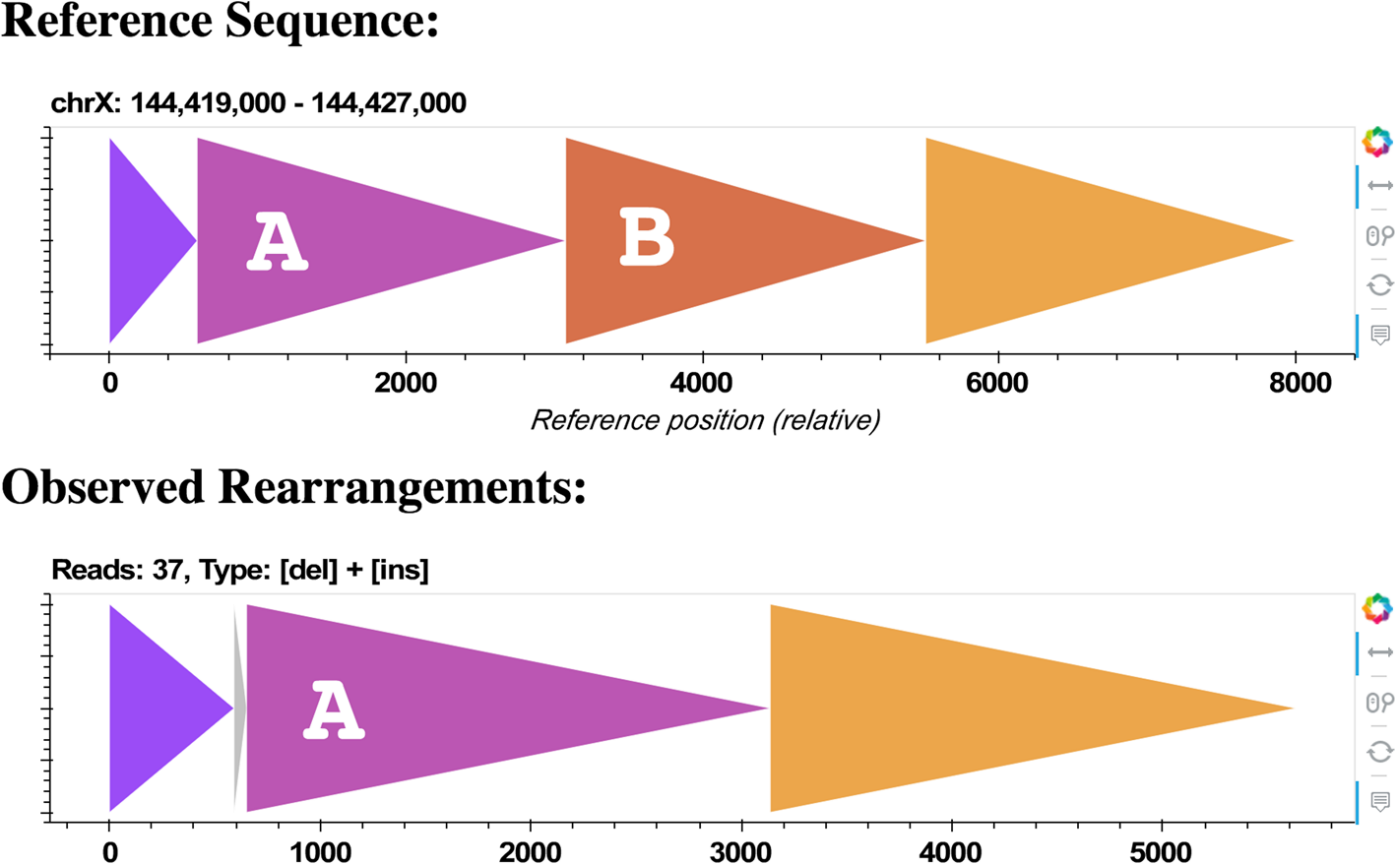
An example output report for NA12878 chrX: 144,419,000 - 144,427,000. This region was found to contain a ∼2k bp deletion (of orange region **B**) and a 60 bp novel insertion (shown in gray). By BLASTing the novel sequence we found that the gray region also originated from chrX. Specifically, it was found at coordinates 144,330,518 - 144,330,577, about 3k bp beyond the region boundaries shown in the report

For the 1674 regions where CORGi’s PacBio and Moleculo results matched, we tabulated all the SV types and compared them against the LUMPY label associated with the site, if available (Table 2). In virtually every case the LUMPY SV call matched the primary feature of the rearrangement reported by CORGi. However, in many regions CORGi detected additional complexities, often in the form of small insertions, duplications, or inversions that flank the larger features of the rearrangement. For example, 50 of the sites called by LUMPY as a deletion were reported by CORGi to be a deletion with small flanking insertion, typically of length 5-20 bp (Fig. 8). In some cases, BLAST was able to determine that the insertions originated from nearby regions (Example shown in Fig. 9). It has been previously observed that up to 35*%* of nonrecurrent SV breakpoints contain small insertions of sequence originating from nearby genomic regions [24], suggesting the formation of these events is related to microhomology-assisted DNA replication [25].

**Table 2:** Categorization of the 1674 genomic sites in NA12878 that were found to have matching CORGi calls in both PacBio and Moleculo data

CORGi SV Call		LUMPY call (if applicable):
Description:	Count:	
DEL	1379	DELETION: 1171
DUP	37	TANDEMDUP: 18
DEL + DUP	3	Complex *†*: 2
DEL + INS	81	DELETION: 50
DEL + INV	8	DELETION: 3, INVERSION: 5
DUP + INS	41	TANDEMDUP: 3, Complex: 1
DUP + INV	5	
INV + INS	3	
MULTI-DEL	18	DELETION: 16
MULTI-DUP	3	TANDEMDUP: 1
DEL + DUP + INV	12	DELETION: 4, INVERSION: 4
MULTI-DEL + INS	5	DELETION: 5
MULTI-DEL + INV	11	DELETION: 2, INVERSION: 6, Complex: 3
MULTI-DUP + DEL	8	Complex: 5
MULTI-DUP + INV	1	TANDEMDUP: 1
NESTED-DUP/DEL ∗	9	
NESTED-DUP/DUP	3	
NESTED-DUP/INV	2	
NESTED-DUP/DEL DEL	2	
NESTED-DUP/DUP DEL	1	Complex: 1
NESTED-DUP/INV DUP	5	

LUMPY calls from short read data are included for the events that intersect LUMPY high-confidence SVs. The prefix “MULTI” in our SV calls indicate multiple occurrences of the same event type in close proximity. *†*: We choose to define complex as any grouping of two or more LUMPY calls within 1kbp of each other. ∗: NESTED-DUP/DEL denotes a duplication such that the added copy of the duplicated region contains within it a deletion. NESTED-DUP/DUP and NESTED-DUP/INV are defined similarly

If both breakpoint coordinates of a rearrangement are within local repetition or low-complexity sequence, it may not be possible to uniquely define their coordinates at base-pair resolution (i.e. there exist multiple pairs of coordinates that would result in identical sequence in the sample’s genome). This often manifests in CORGi’s analysis as alignment matches that are joined during the graph search which have overlapping read coordinates (e.g. Fig. 2 f-h). In our results, only 491 of the 1379 deletion events (35.6%) were found to have breakpoint coordinates that were uniquely defined down to a single base-pair. The breakpoints of the remaining events were found to have a range of possible coordinates, typically 5-20 bp in size (Fig. 8). This observation is corroborated by existing work which found that a majority of nonrecurrent SV breakpoints lie in 2-33 bp microhomologies [26].

**Fig. 10:**
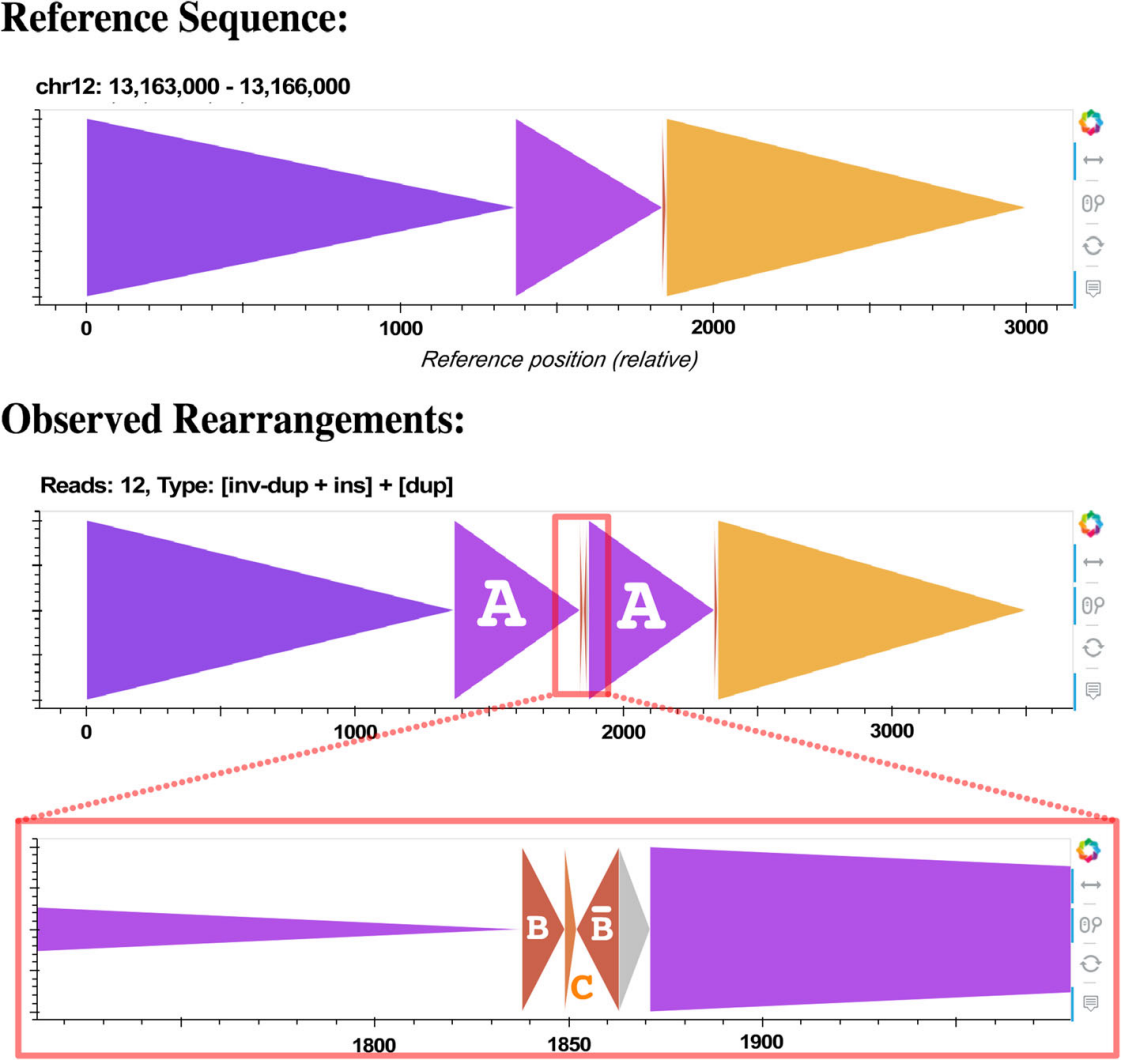
∼400 bp duplication **ABC** with an insertion of $\overline {\mathbf {\texttt {B}}}$**N** (where **N** denotes a novel insertion) in between the two copies. In Table 2 this event was described as “MULTI-DUP + INV.”

In addition, CORGi detected a number of rearrangements involving 3 or more breakpoints that were not variations of the duplication/deletion + small flanking indels as described above. For example, CORGi identified a ∼400 bp tandem duplication in NA12878 chr12 that also featured a smaller inverted duplication and novel insertion placed between the two copies of duplicated sequence (Fig. 10).

### Limitations and future work

CORGi processes a single range of genomic coordinates at a time, thus it is unable to detect very large SVs or SVs that involve multiple chromosomes. Future work could address this limitation via preprocessing the reads and identifying regions outside the specified range that may be involved in the rearrangement. These regions could be incorporated as additional subjects to align against during the exhaustive BLAST realignment, and would need to be integrated into the graph search step.

## Conclusion

In this work we presented CORGi, a tool for detecting and plotting the structure of complex local genomic rearrangements from long-read data via exhaustive alignment and graph search. We demonstrated CORGi’s ability to detect breakpoints in synthetic data, as well as in NA12878 germline from both PacBio and Moleculo long reads. From our analysis of simulated data we observe high sensitivity for reads with ≤5*%* error, and for complex SVs greater than 100 bp in size. From real data we observe many complex patterns that are known to be associated with DNA replication and recombination, such as flanking insertions and breakpoints within local microhomologies. The reports generated by CORGi facilitate detailed investigation of rearrangement structures, allowing users to examine complex SV patterns in long read alignments in greater detail than conventional methods.
